# *Tph2* Gene Expression Defines Ethanol Drinking Behavior in Mice

**DOI:** 10.3390/cells11050874

**Published:** 2022-03-03

**Authors:** Magdalena Zaniewska, Valentina Mosienko, Michael Bader, Natalia Alenina

**Affiliations:** 1 Max-Delbrück-Center for Molecular Medicine, Robert-Rössle-Str. 10, 13125 Berlin, Germany; valentina.mosienko@bristol.ac.uk (V.M.); mbader@mdc-berlin.de (M.B.); 2 Laboratory of Pharmacology and Brain Biostructure, Department of Pharmacology, Maj Institute of Pharmacology, Polish Academy of Sciences, 12 Smętna Street, 31-343 Kraków, Poland; 3 Institute for Biology, University of Lübeck, Ratzeburger Allee 160, 23562 Lübeck, Germany; 4 Charité University Medicine Berlin, Charitéplatz 1, 10117 Berlin, Germany; 5 German Center for Cardiovascular Research (DZHK), Partner Site Berlin, Potsdamer Str. 58, 10785 Berlin, Germany

**Keywords:** central 5-HT, ethanol, mice, raphe nuclei, *Tph2* knockout, *Tph2* transcript

## Abstract

Indirect evidence supports a link between disrupted serotonin (5-hydroxytryptamine; 5-HT) signaling in the brain and addictive behaviors. However, the effects of hyposerotonergia on ethanol drinking behavior are contradictory. In this study, mice deficient in tryptophan hydroxylase 2 (*Tph2*^−/−^), the rate-limiting enzyme of 5-HT synthesis in the brain, were used to assess the role of central 5-HT in alcohol drinking behavior. Life-long 5-HT depletion in these mice led to an increased ethanol consumption in comparison to wild-type animals in a two-bottle choice test. Water consumption was increased in naïve 5-HT-depleted mice. However, exposure of *Tph2*^−/−^ animals to ethanol resulted in the normalization of water intake to the level of wild-type mice. *Tph2* deficiency in mice did not interfere with ethanol-evoked antidepressant response in the forced swim test. Gene expression analysis in wild-type animals revealed no change in *Tph2* expression in the brain of mice consuming ethanol compared to control mice drinking water. However, within the alcohol-drinking group, inter-individual differences in chronic ethanol intake correlated with *Tph2* transcript levels. Taken together, central 5-HT is an important modulator of drinking behavior in mice but is not required for the antidepressant effects of ethanol.

## 1. Introduction

Alcoholism is a chronic relapsing brain disorder defined by a loss of control over drinking despite known health consequences. Current first-line pharmacotherapies include naltrexone, the opioid receptor antagonist, and acamprosate, a drug that modulates glutamate and GABA-ergic signaling [1]. Disulfiram, which is an inhibitor of the acetaldehyde dehydrogenase, an enzyme responsible for ethanol metabolism, is a second-line treatment option [1]. The efficacy of these therapies is low, potentially due to the high diversity in treatment response between alcoholic individuals (for review [2]).

Epidemiological studies demonstrate the correlation between depression and alcohol dependence in humans [3]; however, their casual-connective relations have not been fully established yet [4,5]. Depressive symptoms commonly occur in the period preceding the development of alcohol dependence and very often initiate drug-taking behavior for self-medication, a behavior called autotherapy. Conversely, in many individuals living with alcohol dependence, withdrawal from alcohol induces severe symptoms of depression, which, in turn, significantly increase the risk of relapse. The comorbidity of depression and alcohol abuse further hampers the researchers’ ability to explain the molecular mechanisms underlying these brain disorders and to find effective pharmacotherapy.

Dysregulation of serotonin (5-hydroxytryptamine; 5-HT) neurotransmission has been linked to both depression [6] and ethanol drinking [7]. Studies in humans revealed that low availability of the 5-HT precursor, tryptophan, has been associated with an early onset of alcohol dependence, accompanied by depressive and aggressive symptoms [8]. A decrease in the level of 5-HT or its metabolite, 5-hydroxyindole-acetic acid (5-HIAA), has been found in the cerebrospinal fluid and plasma of some subgroups of alcoholics in the abstinence phase [9,10,11]. Tryptophan depletion studies in healthy individuals demonstrated that low 5-HT enhanced ethanol-induced impulsivity [12], but, in alcoholics, it did not change ethanol consumption or cue-evoked craving [13,14]. On the other hand, increasing 5-HT neurotransmission by selective 5-HT reuptake inhibitors (SSRIs) decreased ethanol drinking in alcoholic individuals, specifically in those with comorbid alcoholism and depression [15,16,17]. However, other studies showed low efficacy of SSRI treatment in alcoholism [18]. Considering the complexity of alcohol use disorder, alcoholics were subgrouped into “lower risk/severity” (type A) and “higher risk/severity” (type B) alcoholics [19], with type B having abnormalities in 5-HT transmission (i.e., a functional polymorphism in 5-HT transporter (5-HTT) gene; for review [7]). Importantly, in type B alcoholics, treatment with SSRIs, such as fluoxetine or sertraline, led to poorer drinking outcomes. However, in type A alcoholics, treatment with sertraline, but not fluoxetine, had a beneficial effect on drinking-related measures [20,21,22]. Such differential response of type A and B alcoholics to SSRIs suggests that further optimization of the classification of alcoholic individuals is needed to achieve the best results in alcoholism treatment using serotoninergic drugs.

Interestingly, preclinical studies have shown that selectively bred alcohol-preferring rats displayed alterations in 5-HT function; however, there is inconsistency in the direction of observed changes [23,24,25,26,27]. Neonatal 5-HT depletion by lesioning the 5-HT fibers by neurotoxin 5,7-dihydroxytryptamine (5,7-DHT) led to decreased ethanol intake in adult rats [28]. The same treatment of adult rats pre-exposed to ethanol either had no effect or enhanced ethanol-drinking behavior [29,30,31,32]. However, behavioral consequences of 5,7-DHT treatment may not only be due to a reduction in 5-HT level, but also to the loss of 5-HT neurons [33].

An alternative approach for assessing the role of central 5-HT neurotransmission that leaves the 5-HT neurons intact is to modulate the activity of enzymes involved in 5-HT synthesis, mainly tryptophan hydroxylase 2 (TPH2). TPH2 is responsible for the conversion of tryptophan to 5-hydroxytryptophan (5-HTP), the rate-limiting step of 5-HT production. *Tph2* mRNA is synthesized within the 5-HT neurons of the brainstem raphe nuclei and can be transported via axonal transport to other brain areas, such as the cortex, hippocampus, striatum, hypothalamus, and cerebellum [34,35,36]. Studies in humans revealed elevated levels of *TPH2* mRNA and protein in the dorsal and median raphe nucleus of alcoholic individuals [37,38]. A study on suicide victims with major depression and alcohol dependence showed enhanced TPH-immunoreactivity only in the dorsal subnucleus of the dorsal raphe [39]. In addition, several single nucleotide polymorphisms in the human *TPH2* gene have been associated with major depression (e.g., R441H; [40,41]) and alcoholism (e.g., intron variant rs1386496; [42,43]); however, other investigators have failed to find the link between *TPH2* gene variants and depression (for review [41]) or alcohol dependence [44,45].

Preclinical studies examining the effects of *Tph2* gene variants on the level of ethanol intake have been inconsistent. For example, mice with a hypofunctional R439H polymorphism in *Tph2* gene (analogous to a human R441H variant), leading to a partial (60–80%) deficiency in 5-HT level in the brain [46,47,48,49], drank more ethanol–sucrose solution compared to wild-type mice [50]. In another study, the R439H *Tph2* knock-in mice drank amounts of ethanol comparable to wild-type animals; however, under aversive conditions, displayed enhanced motivation for ethanol [51]. Low preference for ethanol was reported in two mouse strains, BALB/cJ and DBA/2A, that carry another polymorphism, C1473G (P447R) in the *Tph2* gene, leading to reduction in *Tph2* mRNA and protein levels and about a 15% decrease in 5-HT content in the brain [52,53,54,55].

Mice lacking TPH2 have been generated by several laboratories [56,57,58,59]. Characterization of these mice revealed a number of phenotypes associated with the loss of brain 5-HT, including early postnatal growth retardation, maternal neglect, increased aggression, sleep disturbances, social deficits, reduced anxiety-like behavior, and increased food intake [56,60,61,62]. Evaluation of depression-like behavior did not reveal a clear-cut phenotype. Specifically, depression-like phenotype of *Tph2*-deficient (*Tph2*^−/−^) mice was observed in the forced swim test (FST); however, no difference in behavior compared to wild-type mice was detected in tail suspension and sucrose preference tests (for review [63]). To date, the effects of complete inactivation of the *Tph2* gene on ethanol-drinking behavior have not yet been studied.

Taken together, the data discussed above prompted us to test the hypothesis that the life-long 5-HT deficiency affects the initiation of ethanol drinking and ethanol-induced antidepressant effects in mice. Furthermore, in search of a molecular mechanism linking 5-HT function and ethanol addiction, we analyzed the changes in *Tph2* transcript level in wild-type mice drinking ethanol. In this study, we employed *Tph2*^−/−^ mice that lack central 5-HT throughout the life [56]. We first evaluated alcohol-drinking behavior in *Tph2*^−/−^ mice that had continuous access to ethanol solutions of increasing concentrations in a two-bottle choice procedure. We next evaluated whether the genetic ablation of *Tph2* affected the antidepressant effects of ethanol in the FST. In the second part of the study, we assessed *Tph2* expression in the brain of *Tph2*^+/+^ mice drinking ethanol by quantitative real-time PCR (qPCR). We demonstrate that *Tph2* deficiency in mice enhanced ethanol consumption and that *Tph2* transcript levels correlated with ethanol intake in mice.

## 2. Materials and Methods

### 2.1. Animals

Animal experimental procedures were carried out in accordance with the European Communities Council Directive 2010/63/UE and had been approved by the local animal welfare and ethical review body (Landesamt für Gesundheit und Soziales Berlin (LaGeSo), G 0343/09, date of approval: 24 March 2010). All the efforts were made to minimize animal suffering and to reduce the number of mice used.

Animals were maintained at the animal facility of the MDC (Berlin, Germany). Mice were housed in individually ventilated cages (34 cm × 19 cm × 13 cm; Tecniplast Deutschland, Hohenpeissenberg, Germany) in a colony room maintained at 21 ± 1 °C under a 12-h light–dark cycle (lights on at 7:00 am). Standard rodent chow (0.25% sodium, SSNIFF Spezialitäten, Soest, Germany) and water were available ad libitum. The behavioral experiments were conducted during the light phase (between 8:00 and 16:00) of the light–dark cycle.

Male *Tph2*^−/−^ mice on a C57BL/6 background (F10 generation backcross; *n* = 14) [56,60] and wild-type mice (*Tph2*^+/+^; *n* = 22) weighing 24.31 ± 0.64 g and 23.64 ± 0.33 g, respectively, at the beginning of the experiment (ca. 10 weeks old) were used. To generate C57BL/6 *Tph2*^+/+^ and *Tph2*^−/−^ experimental groups, *Tph2*^+/−^ females were bred with *Tph2*^−/−^ or *Tph2*^+/+^ males. Genotyping was carried out using DNA isolated from the ear snips by multiplex PCR with primers Neo3: 5′-CTGCGCTGACAGCCGGAACAC-3′, TPH34: 5′-AGCTGAGGCAGACAGAAAGG-3′, and TPH54: 5′-CCAAAGAGCTACTCGA CCTACG-3′ to distinguish the *Tph2*-wild-type allele (TPH54/34, 600 bp) from the *Tph2*-knockout allele (TPH54/Neo3; 450 bp).

### 2.2. Behavioral Studies

#### 2.2.1. Drugs

Ethanol (3, 6, or 10%; *v*/*v* diluted from 96% ethyl alcohol; Merck, Darmstadt, Germany) was dissolved in tap water.

#### 2.2.2. Two-Bottle Free-Choice Ethanol Consumption

One week before the experiment, animals (*n* = 36; *Tph2*^+/+^: *n* = 22, *Tph2*^−/−^: *n* = 14) were single housed and habituated to two bottles (one empty and the second filled with water). The position of bottles was changed from the left to the right side to induce seeking behaviors in animals. After 6 days, randomly assigned mice were exposed to a two-bottle free-choice paradigm (water and ethanol; *n* = 26; *Tph2*^+/+^: *n* = 17, *Tph2*^−/−^: *n* = 9) in home cages for 29 days, using increasing concentrations of ethanol solution (3% *v*/*v*, 4 days; 6% *v*/*v*, 4 days; 10% *v*/*v*, 21 days) [64,65,66,67]. Animals exposed to two bottles of water throughout the study were used as controls (*n* = 10; *Tph2*^+/+^: *n* = 5, *Tph2*^−/−^: *n* = 5). Every two days, the bottles were refilled with ca. 14 mL of fresh solutions and the position of the bottles was alternated to control for side preference. Liquid (alcohol and water) intake was measured by weighing the bottles in 2-day trials (after 48 h) for 28 days and on the final day of experiment (day 29) after 24 h. Ethanol intake was calculated as g of pure ethanol per kg of body weight per 1 or 2 days (g/kg). Preference was calculated as g of ethanol solution consumed within 1 or 2 days per total amount of liquid (%). Cumulative ethanol intake was calculated as g of pure ethanol per kg of body weight within 29 days (g/kg/29 d). We also calculated the average intake of ethanol (g/kg/d), preference (%), and average total liquid intake (mL of total liquid (water or water + ethanol) consumed per kg of body weight per day (mL/kg/d)) during the initial 4 days of each ethanol concentration.

#### 2.2.3. Forced Swim Test (FST)

Immediately after the end of the last ethanol exposure day, mice were introduced to the FST. As previously described [60,68,69], mice were individually placed in a plastic beaker (24 cm high and 17.5 cm in diameter) filled with 18 cm of water (23 ± 1 °C). The following parameters were scored manually in 2-min intervals for 6 min (the “pretest”: 0–2 min, “test”: 2–6 min): latency to the first immobility episode, immobility, swimming, and climbing. Latency was defined as the time that elapsed between placing the mouse in the beaker and the first immobility episode. A mouse was rated immobile if it was floating on the water, making only movements necessary to keep its head above water. Swimming was defined as horizontal movements of four legs or just the hindlimbs. Climbing was scored when the mouse was making struggling vertical movements of the forepaws, directed against the wall of the beaker [70]. After the test, mice were then removed from the water, dried with towels, and placed in a warm enclosure before they were returned to their home cages. The beakers were emptied and cleaned between mice.

### 2.3. Molecular Analyses

#### 2.3.1. *Tph2* Gene Expression Analysis in “High Ethanol-Drinking” and “Low Ethanol-Drinking” Mice

For the *Tph2* gene expression analysis, only the brain tissue from *Tph2*^+/+^ mice (*n* = 22; water group: *n* = 5, or water/ethanol group: *n* = 17) was taken. First, the *Tph2* expression level in the whole group of *Tph2*^+/+^ mice drinking ethanol (*n* = 17) was compared with water-drinking mice (*n* = 5).

Since there was considerable variability in the level of ethanol intake and preference for ethanol among *Tph2*^+/+^ mice during the experiment, the level of *Tph2* expression was then analyzed with respect to the animals’ ethanol intake levels in two drinking periods: during the initial days of ethanol drinking (i.e., the first 12 days of drinking different concentrations of ethanol) or during the last week of access to ethanol (period of stabilized ethanol drinking). For each period (analyzed separately), the average ethanol intake (g/kg/d) was calculated for each animal. In addition, an average preference (%) was calculated for each animal for each of the tested periods. The average ethanol intake scores were used to divide mice into “high ethanol-drinking” (*n* = 9) and “low ethanol-drinking” (*n* = 8) animals using a median split [71], i.e., animals with values of ethanol intake below the median were classified as low drinkers, while animals’ scores equal to or greater than the median were considered as high drinkers. For each analyzed period, the transcript levels of “high ethanol-drinking” mice were compared with those of “low ethanol-drinking” animals.

Such analysis between ethanol intake levels in mice, classified as high- or low-ethanol drinkers at different drinking periods, and *Tph2* expression was aimed at examining whether the inter-individual differences in *Tph2* mRNA levels were notable prior to ethanol exposure (assuming that ethanol did not affect *Tph2* expression) or were a result of the long-term exposure of animals to alcohol.

#### 2.3.2. Sample Preparation

Immediately after the end of the FST, animals were sacrificed under non-stressful conditions by quick decapitation and the brains were rapidly dissected and chilled in ice-cold saline. Raphe nuclei of the brainstem, hippocampus (including the dentate gyrus, CA3, CA2, CA1, and subiculum), and the prefrontal cortex were dissected according to the Mouse Brain Atlas [72]. Brain tissue was preserved in RNAlater RNA Stabilization Reagent (QIAGEN, Erlangen, Germany), frozen on dry ice, and stored at −80 °C to prevent degradation of RNA.

Total RNA was isolated from the above brain areas using a phenol-based method (TRIzol Reagent; Invitrogen, Darmstadt, Germany). Further RNA cleanup and on-column DNase digestion were performed using RNeasy Mini Kit (QIAGEN, Hilden, Germany) according to the manufacturer’s instructions. RNA quantification and purity were assessed using NanoDrop^TM^ spectrophotometer ND-1000 (Peqlab, Erlangen, Germany). A total of 1 µg of isolated RNA was reverse transcribed using random hexamer primers and Moloney Murine Leukemia Virus Reverse Transcriptase (M-MLV RT; Invitrogen). The samples were then processed for qPCR to assess the expression of *Tph2.*

#### 2.3.3. Real-Time PCR

Gene expression analysis was performed by qPCR using ABI Prism 7900 HT Fast RT-PCR System (Applied Biosystems, Darmstadt, Germany) with GoTaq qPCR Master Mix (A600A PROMEGA, Madison, WI, USA), 22.5 ng of cDNA, and the following primers: *Tph2* (NM_173391.3): SAB-RT_mTPH2_FW: 5′-GTCAATTACCCGTCC CTTCTC-3′; SAB-RT_mTPH2_REV: 5′-TTATTCAAGGCATCACACACTG-3′ (product length: 138 bp); a housekeeping gene, TATA box binding protein (*Tbp*; NM_013684.3): forward 5′-CCCTATCACTCCTGCCACACC-3′; reverse 5′-CGAAGTGCAATGGTCTTT AGGTC-3′ (product length: 117 bp). For each tested brain region, standard curves with serial dilutions of cDNAs were performed to analyze the efficiency of the primers. Samples were run in triplicates in 384-well plate formats. A negative control lacking cDNA was included for each primer pair. All the data were normalized to the mRNA level of *Tbp*. To show differential expression of *Tph2* in the brain of control mice, the abundance of *Tph2* mRNA was calculated according to the following equation:

(1) $$abundance=2^{-C_{T}}.$$

In the analysis of the effects of ethanol on *Tph2* transcript level, a comparative cycle threshold (*C*_T_) method ($2^{-\Delta \Delta C_{T}}$ method; [73]) was used. Results are shown as fold change ($2^{-\Delta \Delta C_{T}})$ means of the gene of interest.

### 2.4. Statistical Analyses

Data are expressed as the means (± SEM) or median and percentile range. The normality of data distribution was tested by the Shapiro–Wilk normality test. After checking all the assumptions (i.e., normal distribution, equality of variance), the appropriate statistical tests were applied. Student’s *t*-test for independent samples was used to study comparisons between means representing changes from the control values (e.g., *Tph2* transcript level analyses). In the case of the occurrence of unequal variances, Student’s *t*-test with Welch correction was used. In the case of the data in which the data distribution was not normal, a Mann–Whitney U test was used. A two-way repeated-measures analysis of variance (ANOVA) was used to analyze the liquid (or water) intake measurements (factors: genotype, treatment (ethanol), ethanol concentration). One-way repeated-measures ANOVA was used to process ethanol consumption and preference data (factors: genotype, day) of the 2-day measurements performed over 28 days of the experiment. Two-way ANOVA was used to analyze the FST data (factors: genotype, treatment (ethanol)). One-way ANOVA was used to verify differences in *Tph2* transcript level between different brain regions, and between “high ethanol-drinking” and “low ethanol-drinking” mice. ANOVA was followed by a post hoc Newman–Keuls test. All comparisons were made with an experiment type I error rate (α) set at *p* < 0.05.

## 3. Results

### 3.1. Tph2 Deficiency Leads to Increased Ethanol Consumption in Mice

We first evaluated the ethanol drinking behavior in *Tph2*^−/−^ and *Tph2*^+/+^ mice with continuous access to increasing ethanol concentrations in a two-bottle choice procedure.

The body weight of *Tph2*^−/−^ mice was similar to *Tph2*^+/+^ animals at the beginning of the experiment (*Tph2*^+/+^: 23.64 ± 0.33 g, *Tph2*^−/−^: 24.31 ± 0.64 g; t = 1.03, df = 34, *p* = 0.31, η^2^ = 0.03).

**Figure 1:**
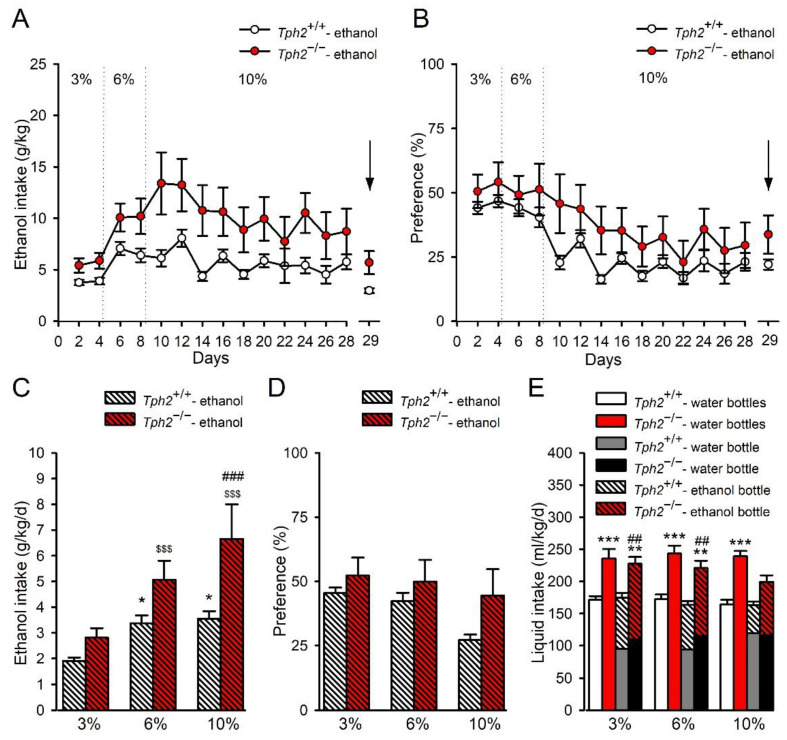
Ethanol consumption in *Tph2*^−/−^ deficient mice in a two-bottle choice test. Mice were exposed to a two-bottle (water and ethanol) free-choice paradigm in home cages for 29 days (ethanol: 3% *v*/*v*, 4 days; 6% *v*/*v*, 4 days; 10% *v*/*v*, 21 days). Animals that were given access to two bottles of water were used as controls. Liquid (alcohol and water) intake was measured by weighing the bottles in 2-day trials for 28 days. On the final day of ethanol drinking (day 29), liquid intake was measured after 24 h, and mice were introduced to the forced swim test (FST). Immediately after the test, animals were killed by decapitation (black arrow) and the brain tissue was collected. The following parameters were measured: (**A**) ethanol intake—g of pure ethanol consumed per kg of body weight per 1 or 2 days, (g/kg); (**B**) preference—g of ethanol solution consumed per total amount of liquid, %; (**C**) average intake of ethanol during the first 4 days of each concentration of ethanol, (g/kg/d); (**D**) preference during 4 days of each concentration of ethanol, %; (**E**) average intake of total liquid during 4 days of each concentration of ethanol—mL of total liquid (water or water + ethanol) consumed per kg of body weight per day, (mL/kg/d). The data are expressed as the means (± SEM) of the data from 5–17 mice/group. (**A**) genotype effect: *p* < 0.01, day effect: *p* < 0.001; (**B**) day effect: *p* < 0.001; (**C**) post hoc Newman–Keuls: * *p* < 0.05 vs. *Tph2*^+/+^- ethanol (3%); ^$$$^ *p* < 0.001 vs. *Tph2*^−/−^- ethanol (3%); ^###^ *p* < 0.001 vs. *Tph2*^+/+^- ethanol (10%); (**D**) ethanol dose effect: *p* < 0.001 10% ethanol vs. 3% or 6%; (**E**) liquid intake: post hoc Newman–Keuls: ** *p* < 0.01, *** *p* < 0.001 vs. appropriate *Tph2*^+/+^- water group; ^##^ *p* < 0.01 vs. appropriate *Tph2*^+/+^- ethanol group. Water intake: genotype effect: *p* < 0.01, treatment effect: *p* < 0.001.

ANOVA analyses revealed that the effect of *Tph2* deficiency on ethanol consumption with a two-bottle choice paradigm did not change according to drinking days (no effect of genotype x day interaction (F(13,312) = 1.40, *p* = 0.16, ηp^2^ = 0.055); Figure 1A). However, there was a significant effect of genotype (F(1,24) = 9.58, *p* = 0.0049, ηp^2^ = 0.29) and day (F(13,312) = 6.03, *p* < 0.001, ηp^2^ = 0.20) on the level of ethanol consumption during the chronic exposure of *Tph2*^−/−^ mice to ethanol (Figure 1A), indicating that both factors, independent of one another, affected ethanol intake in mice.

There was a significant increase in cumulative (during 29 days) ethanol intake in *Tph2*^−/−^ mice in comparison to *Tph2*^+/+^ mice (*Tph2*^+/+^: 80.53 ± 4.57 (g/kg/29 d), *Tph2*^−/−^: 139.45 ± 24.80 (g/kg/29 d); t = 2.34, df = 8.55, *p* = 0.046, η^2^ = 0.39).

ANOVA analyses showed that the effect of *Tph2* deficiency in mice on preference for ethanol did not change depending on ethanol drinking days (no effect of genotype x day interaction (F(13,312) = 0.92, *p* = 0.53, ηp^2^ = 0.037); Figure 1B). However, there was a significant effect of drinking days (F(13,312) = 15.61, *p* < 0.001, ηp^2^ = 0.39), but not genotype (F(1,24) = 3.79, *p* = 0.063, ηp^2^ = 0.14), on the preference for ethanol (Figure 1B), indicating that preference for ethanol, independent of genotype, was changed throughout days.

Further data analysis revealed that the effect of *Tph2* deficiency on the average intake of ethanol during 12 initial days (4 initial days of each ethanol concentration) of ethanol drinking changed according to different ethanol concentrations (a significant effect of genotype x ethanol dose interaction (F(2,48) = 4.76, *p* = 0.013, ηp^2^ = 0.17), genotype (F(1,24) = 9.68, *p* = 0.0048, ηp^2^ = 0.29), and ethanol concentration (F(2,48) = 29.94, *p* < 0.001, ηp^2^ = 0.56); Figure 1C). Post hoc Newman–Keuls analysis showed that consumption of 6 and 10% ethanol in *Tph2*^+/+^ mice was significantly increased compared to the 3% ethanol concentration by 76 and 86%, respectively (*p* = 0.018 and 0.012, respectively; Figure 1C). In addition, *Tph2*^−/−^ mice exhibited increased by 80% consumption of 6% ethanol and increased by 136% consumption of 10% ethanol in comparison to the consumption of 3% ethanol (*p* = 0.00048 and 0.00013, respectively; Figure 1C). Importantly, *Tph2*^−/−^ mice drank significantly more 10% ethanol than *Tph2*^+/+^ animals (*p* = 0.00046; Figure 1C).

ANOVA analyses showed that the effect of depletion of brain 5-HT in *Tph2*^−/−^ mice on preference for ethanol during the first 12 days of exposure did not change depending on ethanol concentrations (no effect of genotype x ethanol dose interaction (F(2,48) = 1.96, *p* = 0.15, ηp^2^ = 0.076; Figure 1D). However, the analysis demonstrated a significant effect of ethanol concentration (F(2,48) = 10.75, *p* = 0.00014, ηp^2^ = 0.31), but not genotype (F(1,24) = 2.87, *p* = 0.10, ηp^2^ = 0.11), on the preference for ethanol (Figure 1D). Newman–Keuls test of the main effect of ethanol concentration revealed a significant reduction in preference in all animals drinking 10% ethanol compared to mice drinking 3% (*p* = 0.00014) or 6% (*p* = 0.00024) ethanol (Figure 1D).

As estimated, during 12 initial days of ethanol drinking, the effect of *Tph2* deficiency in mice significantly altered average liquid (ethanol + water) intake according to treatment and ethanol concentration (a significant effect of genotype x treatment (ethanol) x ethanol dose interaction (F(2,64) = 3.81, *p* = 0.027, ηp^2^ = 0.11); Figure 1E). There was a significant effect of genotype (F(1,32) = 39.51, *p* < 0.001, ηp^2^ = 0.55) and ethanol concentration (F(2,64) = 8.71, *p* = 0.00045, ηp^2^ = 0.21), but no effect of ethanol treatment (F(1,32) = 1.84, *p* = 0.19, ηp^2^ = 0.054) on total liquid consumption (Figure 1E). Newman−Keuls analysis demonstrated that, in comparison to water-drinking *Tph2*^+/+^ animals, a significant increase in average liquid intake was reported in *Tph2*^−/−^ mice with access to two bottles of water during the first 4 days of exposure to 3% (*p* = 0.00097), 6% (*p* = 0.00038) and 10% (*p* = 0.00028) ethanol. Importantly, in *Tph2*^−/−^ mice drinking ethanol and water, total liquid intake was enhanced only during the four initial days of exposure to 3% (*p* = 0.0035) and 6% (*p* = 0.0071), but not to 10% (*p* = 0.12), ethanol compared to appropriate *Tph2*^+/+^ water groups (Figure 1E). Total liquid consumption in *Tph2*^−/−^ mice exposed to 3–6%, but not to 10%, ethanol was significantly higher than that of *Tph2*^+/+^ animals drinking ethanol (*p* = 0.0030 and *p* = 0.0040, respectively; Figure 1E).

As estimated during the 12 initial days of ethanol drinking, the effect of 5-HT depletion in *Tph2*^−/−^ mice on average water intake did not change according to treatment or ethanol concentration (no effect of genotype x treatment (ethanol) x ethanol dose interaction (F(2,64) = 1.28, *p* = 0.28, ηp^2^ = 0.038); Figure 1E). However, there was a significant effect of genotype (F(1,32) = 9.89, *p* = 0.0036, ηp^2^ = 0.24) and ethanol treatment (F(1,32) = 55.23, *p* < 0.001, ηp^2^ = 0.63), but no effect of ethanol concentration (F(2,64) = 0.92, *p* = 0.40, ηp^2^ = 0.028), on water consumption (Figure 1E). This analysis implied that both genotype and ethanol treatment, independent of the other factor, influenced the average water intake in mice, i.e., *Tph2* deficiency increased, whereas ethanol exposure reduced, water intake (Figure 1E).

Overall, increasing the ethanol concentrations (6–10%) enhanced ethanol intake in both genotypes, with the highest (10%) concentration inducing a significantly higher ethanol consumption in *Tph2*^−/−^ compared to *Tph2*^+/+^ mice. Continuous access to 10% ethanol decreased preference for ethanol, independent of the genotype group. During the first days of exposure to each ethanol concentration, average liquid consumption was increased in *Tph2*^−/−^ mice that were given access to two bottles of water, while, in *Tph2*^−/−^ mice drinking ethanol, total liquid intake was enhanced only when mice were exposed to lower (3–6%) ethanol concentrations. Continuous access to ethanol reduced average water intake in both genotypes.

### 3.2. Ethanol Exposure in Tph2^−/−^ Mice Induces Antidepressant Effects

**Figure 2:**
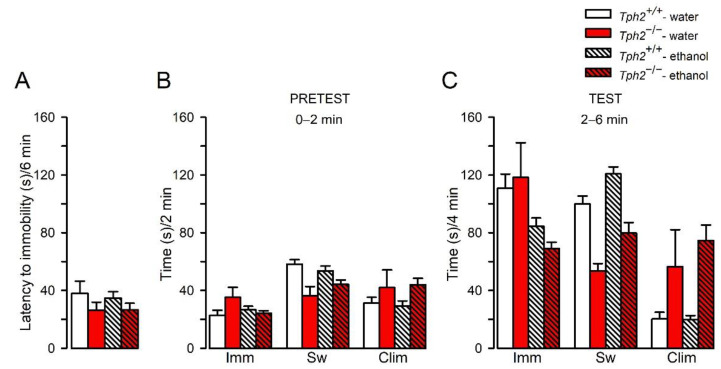
Effects of ethanol consumption in *Tph2*^−/−^ mice on the behavior in the FST. Following 29 days of exposure to the increasing concentrations of ethanol, the animals were individually placed in a beaker filled with water, and the following parameters were measured in 2-min intervals for 6 min: (**A**) latency to immobility, and immobility (Imm), swimming (Sw), and climbing (Clim) during (**B**) “PRETEST”: 0–2 min and (**C**) “TEST”: 2–6 min. The results are shown as the means (± SEM) of data from 5–17 mice/group. (**B**) “Sw”: genotype effect: *p* < 0.01; “Clim”: genotype effect: *p* < 0.05. (**C**) “Imm”: treatment effect: *p* < 0.001; “Sw”: genotype effect: *p* < 0.001, treatment effect: *p* < 0.01; “Clim”: genotype effect: *p* < 0.001.

We next evaluated whether the genetic ablation of *Tph2* affected the antidepressant effects of ethanol. As measured in the FST, the effect of *Tph2* deficiency in mice on the latency to the first immobility episode did not change according to ethanol exposure (no effect of genotype x treatment interaction (F(1,32) = 0.069, *p* = 0.79, ηp^2^ = 0.0021); Figure 2A). There was no effect of genotype (F(1,32) = 2.29, *p* = 0.14, ηp^2^ = 0.067) or treatment (F(1,32) = 0.057, *p* = 0.81, ηp^2^ = 0.0018) on latency parameter (Figure 2A).

Two-way ANOVA analysis of the immobility time during the first 2-min (“pretest”) measurement period revealed a significant effect of genotype x treatment interaction (F(1,32) = 4.17, *p* = 0.049, ηp^2^ = 0.12), but no effect of genotype (F(1,32) = 1.91, *p* = 0.18, ηp^2^ = 0.056) or treatment (F(1,32) = 0.99, *p* = 0.33, ηp^2^ = 0.030), on immobility. However, the post hoc Newman–Keuls showed no significant changes between the genotypes (*p* > 0.05; Figure 2B).

Statistical analyses demonstrated that the effect of *Tph2* deficiency on swimming behavior in the FST during an initial 0–2 min did not alter according to ethanol treatment (no effect of genotype x treatment interaction (F(1,32) = 1.87, *p* = 0.18, ηp^2^ = 0.055); Figure 2B). However, there was a significant effect of genotype (F(1,32) = 11.44, *p* = 0.0019, ηp^2^ = 0.26), but not ethanol treatment (F(1,32) = 0.13, *p* = 0.72, ηp^2^ = 0.0041), on swimming during the 0–2-min measurement (Figure 2B). This analysis indicated that *Tph2* gene inactivation reduced swimming behavior in mice.

The effect of genetic inactivation of *Tph2* in mice on climbing behavior during the 0–2-min time interval did not alter depending on the exposure to ethanol (no effect of genotype x treatment interaction (F(1,32) = 0.12, *p* = 0.73, ηp^2^ = 0.0037); Figure 2B). However, there was a significant effect of genotype (F(1,32) = 4.47, *p* = 0.042, ηp^2^ = 0.12), but not ethanol treatment (F(1,32) = 0.0001, *p* = 0.99, ηp^2^ = 0.000002), on climbing behavior during the 0–2-min measurement (Figure 2B). This indicated that *Tph2* deficiency enhanced climbing behavior in mice.

Throughout the “test” (during a 2–6-min measurement), the effect of *Tph2* deficiency on the immobility time did not change according to ethanol intake (no effect of genotype x treatment interaction (F(1,32) = 1.20, *p* = 0.28, ηp^2^ = 0.036; Figure 2C). A significant effect of ethanol exposure (F(1,32) = 13.53, *p* = 0.00086, ηp^2^ = 0.3), but not genotype (F(1,32) = 0.14, *p* = 0.71, ηp^2^ = 0.0044), on the immobility parameter was detected (Figure 2C), implying that ethanol consumption reduced immobility in both genotypes.

The analyses revealed that the effect of *Tph2* deficiency on swimming time did not alter according to ethanol exposure (no effect of genotype x treatment interaction (F(1,32) = 0.13, *p* = 0.72, ηp^2^ = 0.004; Figure 2C). However, a significant effect of genotype (F(1,32) = 37.24, *p* = 0.000001, ηp^2^ = 0.54) and ethanol (F(1,32) = 10.90, *p* = 0.0024, ηp^2^ = 0.25) on swimming were found (Figure 2C). The analysis indicated that *Tph2* deficiency reduced, whereas ethanol consumption significantly increased, swimming parameter.

As for the climbing behavior, the effect of *Tph2* deficiency in mice did not change according to ethanol consumption (no effect of genotype x treatment interaction (F(1,32) = 0.82, *p* = 0.37, ηp^2^ = 0.025); Figure 2C). However, a significant effect of genotype (F(1,32) = 19.23, *p* = 0.00012, ηp^2^ = 0.38), but not treatment (F(1,32) = 0.72, *p* = 0.40, ηp^2^ = 0.022), on climbing time was reported (Figure 2C), suggesting that *Tph2* deficiency, by itself, elevated climbing behavior.

Taken together, *Tph2* deficiency induced a decrease in swimming and an increase in climbing behavior, without alterations in immobility time. Depletion of brain 5-HT had no effect on ethanol-induced antidepressant phenotype measured as a decrease in immobility time and an increase in swimming in the FST in mice.

### 3.3. Wild-Type “High Ethanol-Drinking” Mice Display Alterations in Tph2 Expression Pattern Compared to “Low Ethanol-Drinking” Animals

**Figure 3:**
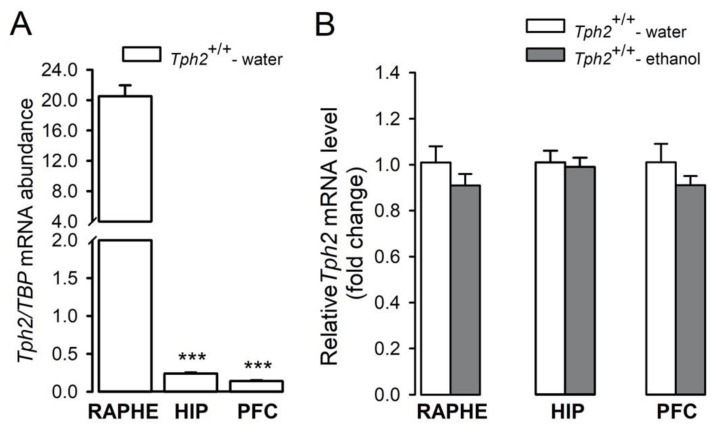
Effects of chronic ethanol consumption on the transcript levels of *Tph2* in the selected mouse brain structures. *Tph2*^+/+^ mice were exposed to increasing concentrations of ethanol (3–10%) for 29 days. Animals that were given access to two bottles of water were used as controls. On the last day of ethanol drinking, mice were introduced to the FST and, after the test, they were immediately killed by decapitation. The *Tph2* transcript level was analyzed in the raphe nuclei (RAPHE), hippocampus (HIP), and prefrontal cortex (PFC) by RT-qPCR. All the data were normalized to a housekeeping gene, TATA box binding protein (*Tbp*). To show differential expression of *Tph2* in the brain of control mice, the abundance of *Tph2* mRNA was calculated (**A**). In the analysis of the effects of ethanol on *Tph2* transcript level (**B**), results were shown as fold change ($2^{-\Delta \Delta C_{T}}$) to control mice. Data are presented as means of data from 5–17 mice/group. (**A**): post hoc Newman–Keuls: *** *p* < 0.001 vs. *Tph2*^+/+^-water (RAPHE).

In search of a mechanistic link between 5-HT synthesis and alcohol intake in mice, we investigated whether *Tph2* expression is affected by ethanol exposure in wild-type animals. One-way ANOVA analysis showed that the level of *Tph2* transcripts in the raphe nuclei, hippocampus, and prefrontal cortex of control mice was significantly different (F(2,12) = 199.99, *p* < 0.001, ηp^2^ = 0.97; Figure 3A). Newman–Keuls analysis revealed that the amount of *Tph2* mRNA in the hippocampus and prefrontal cortex in control animals was 86- and 145-fold, respectively, lower than in the raphe nuclei (*p* = 0.00017 and *p* = 0.00019, respectively; Figure 3A). There was no difference in *Tph2* transcript levels between the hippocampus and prefrontal cortex of the wild-type mice (*p* = 0.94; Figure 3A).

Based on the data obtained from all *Tph2*^+/+^ mice drinking ethanol, no change in the expression level of *Tph2* gene was observed in the raphe nuclei (t = 1.08, df = 20, *p* = 0.29, η^2^ = 0.055), hippocampus (U = 34.50, *p* = 0.55), or prefrontal cortex (t = 1.18, df = 20, *p* = 0.25, η^2^ = 0.065) compared to mice drinking water (Figure 3B).

We next analyzed the *Tph2* expression level in the raphe nuclei, hippocampus, and prefrontal cortex of mice divided on “low ethanol-drinking” and “high ethanol-drinking” animals based on the average level of ethanol intake during the initial 12 days and the last week of access to ethanol.

**Figure 4:**
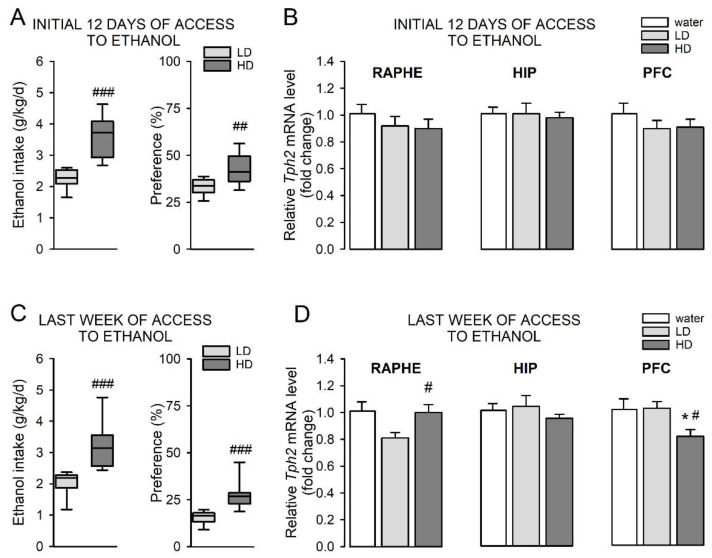
Transcript level of *Tph2* in the brain of “high ethanol-drinking” and “low ethanol-drinking” mice over different experimental periods. *Tph2*^+/+^ mice were exposed to increasing concentrations of ethanol (3–10%) for 29 days. Animals that were given access to two bottles of water were used as controls. On the last day of ethanol drinking, mice were introduced to the FST and, after the test, they were immediately killed by decapitation. The *Tph2* transcript level was analyzed in the raphe nuclei (RAPHE), hippocampus (HIP), and prefrontal cortex (PFC) by RT-qPCR. For each investigated period—(**A**,**B**) an initial 12 days of access to ethanol and (**C**,**D**) the last week of access to ethanol—mice were divided into “high ethanol-drinking” (HD) and “low ethanol-drinking” (LD) mice based on individual differences in the average ethanol intake levels. For each analyzed period, average ethanol intake (g/kg/d) and average preference for ethanol (%) were calculated for HD and LD groups (**A**,**C**). The *Tph2* transcript levels were then compared between HD and LD mice (**B**,**D**). As a control, the *Tph2* mRNA level for mice drinking water was used. (**A**,**C**): Data are shown as box plots, in which the horizontal line depicts the median, and vertical boxes and whiskers correspond to the percentile range. *n* = 8–9 mice/group. (**B**,**D**): All RT-qPCR data were normalized to a housekeeping gene, TATA box binding protein (*Tbp*), and represent fold change ($2^{-\Delta \Delta C_{T}}$) means of data from 5–9 mice/group. (**A**): *t*-test: ^##^ *p* < 0.01, ^###^ *p* < 0.001 vs. LD. (**C**): Mann–Whitney U test: ^###^ *p* < 0.001 vs. LD. (**D**): RAPHE: post hoc Newman–Keuls: ^#^ *p* < 0.05 vs. LD; PFC: post hoc Newman–Keuls: * *p* < 0.05 vs. water, ^#^ *p* < 0.05 vs. LD.

During the first 12 days of drinking different concentrations of ethanol, “high ethanol-drinking” mice displayed an increased average ethanol intake (*t* = 5, df = 15, *p* = 0.0002, η^2^ = 0.63) and preference (*t* = 2.97, df = 15, *p* = 0.0095, η^2^ = 0.37) compared to “low ethanol-drinking” animals (Figure 4A). One-way ANOVA analyses of *Tph2* expression levels revealed that, in this period, there was no difference in the *Tph2* transcript level in the raphe nuclei (F(2,19) = 0.58, *p* = 0.57, ηp^2^ = 0.058), hippocampus (F(2,19) = 0.065, *p* = 0.94, ηp^2^ = 0.0068), and prefrontal cortex (F(2,19) = 0.67, *p* = 0.52, ηp^2^ = 0.066) in these groups of mice (Figure 4B).

The analysis of ethanol intake data from the last week of the ethanol exposure showed that “high ethanol-drinking” mice exhibited an increase in average ethanol intake (U = 0, *p* < 0.0001) and preference (U = 1, *p* = 0.0002) compared to “low ethanol-drinking” animals (Figure 4C). The one-way ANOVA analysis revealed that the *Tph2* transcript level in the raphe nuclei of “high ethanol-drinking” and “low ethanol-drinking” mice was significantly changed (F(2,19) = 3.83, *p* = 0.04, ηp^2^ = 0.29; Figure 4D). Post hoc Newman–Keuls analysis revealed that the expression level of *Tph2* in the raphe nuclei of mice that exhibited high levels of ethanol intake was significantly increased compared to “low ethanol-drinking” mice (*p* = 0.037; Figure 4D). Importantly, the raphe nuclei *Tph2* mRNA levels in high or low ethanol-drinking mice did not differ compared to control mice (*p* = 0.93 or *p* = 0.076, respectively; Figure 4D).

No change in the level of *Tph2* mRNA was observed in the hippocampus of mice with high and low levels of ethanol consumption during the last week of ethanol exposure (F(2,19) = 0.80, *p* = 0.46, ηp^2^ = 0.078; Figure 4D).

There was a significant change in *Tph2* expression in the prefrontal cortex of “high ethanol-drinking” and “low ethanol-drinking” mice during the last week of ethanol consumption (F(2,19) = 4.96, *p* = 0.019, ηp^2^ = 0.34; Figure 4D). Post hoc Newman–Keuls analysis revealed that the expression level of *Tph2* in the prefrontal cortex of mice that exhibited high levels of ethanol intake was significantly decreased compared to mice drinking water (*p* = 0.022) or animals that consumed low levels of ethanol (*p* = 0.049) (Figure 4D). Importantly, the prefrontal cortex *Tph2* mRNA levels in “low ethanol-drinking” mice did not differ compared to control mice (*p* = 0.95; Figure 4D).

Overall, the amount of *Tph2* mRNA in the raphe nuclei of control animals was almost two orders of magnitude higher than the level of *Tph2* mRNA in the hippocampus and prefrontal cortex. Division of mice into “high ethanol-drinking” and “low ethanol-drinking” animals showed that, in high ethanol drinkers, the *Tph2* expression level was increased in the raphe nuclei and reduced in the prefrontal cortex compared to low ethanol drinkers.

## 4. Discussion

This study revealed that *Tph2* deficiency in mice induced an increase in ethanol consumption in a two-bottle choice test. *Tph2*^−/−^ mice that were given continuous access to water only consumed more of that liquid compared to wild-types. However, following ethanol exposure, water consumption decreased to the level of *Tph2*^+/+^ animals with access to water and ethanol. Independent of ethanol treatment, *Tph2* deficiency led to the reduction in swimming, but facilitated climbing behavior in the FST, without changes in immobility time. 5-HT depletion in *Tph2*^−/−^ mice did not influence the development of an antidepressant phenotype (reflected by the reduction of immobility time and an increase in swimming) in the FST following 29 days of ethanol consumption. Evaluation of mRNA levels by qPCR in wild-type mice drinking water revealed, besides strong *Tph2* expression in the raphe nuclei, very low levels of *Tph2* transcripts in the 5-HT projection areas, hippocampus, and prefrontal cortex. Overall, chronic ethanol consumption in wild-type mice did not affect *Tph2* expression in these brain regions. However, in mice drinking high levels of ethanol during the last week of ethanol exposure, a higher *Tph2* expression in the raphe nuclei and lower level of *Tph2* mRNA in the prefrontal cortex were noted in comparison to “low ethanol-drinking” mice.

In our study, we employed a 29-day two-bottle choice procedure, in which animals had continuous access to water and increasing ethanol concentrations, a model often used by many research groups to evaluate the effect of genetic variations on drug response [64,65,66]. Importantly, animals that we tested consumed less ethanol compared to what is reported in the literature in the same paradigm (daily ethanol intake for a 10% ethanol solution: 3 g/kg/d; present study vs. 8 g/kg/d or higher; [65,66,67]). Nevertheless, the protocol used in the present work allowed us to demonstrate the ethanol-induced antidepressant response in the FST in the wild-type mice, as evidenced by decreased immobility time and increased swimming. Our findings in the FST are in line with a previous study that demonstrated antidepressant properties of ethanol following a 28-day voluntary 10% ethanol drinking [74].

The finding of enhanced ethanol drinking in *Tph2*^−/−^ mice is in line with previous studies showing that mice expressing the hypofunctional R439H allele for the *Tph2* gene consumed more ethanol–sucrose solution, had increased preference for ethanol, and reduced sensitivity to ethanol compared to the wild-type animals [50]. However, another study found that the R439H *Tph2* knock-in mice exhibited no change in ethanol intake relative to the control animals, but, only under aversive conditions, motivation for ethanol consumption increased [51]. While the methodological differences (e.g., the use of sucrose-fading procedure [50]; or the introduction of a 4-day withdrawal phase [51]) may account for the observed inconsistencies in studies of R439H *Tph2* mutant mice, the variability in drinking-related outcomes between these animals and *Tph2*^−/−^ mice is likely to be related to the extent of the brain 5-HT depletion. Indeed, R439H mutation in *Tph2* gene resulted in partial (60–80%) reduction in 5-HT levels in the frontal cortex, amygdala, striatum, and hippocampus [46,47,48,49]. In contrast, complete inactivation of the *Tph2* gene in mice used in the current study evoked severe (>98%) 5-HT depletion in all the tested brain regions, including those analyzed in R439H *Tph2* mice [56]. Hence, the brain area which drives 5-HT-dependent alcohol drinking remains unclear. Chemogenetic inhibition of specific 5-HT pathways originating from the raphe nuclei may be instrumental in identifying such brain areas.

In the FST, ethanol-naïve *Tph2*^−/−^ mice displayed a decrease in swimming, confirming previous data showing that swimming is a 5-HT-related parameter [75]. A reduction in swimming time in *Tph2*^−/−^ mice may suggest that these animals developed a modest depression-like state. These results partially support our previous study, in which *Tph2*^−/−^ mice showed a reduced latency to immobility and increased immobility time in the FST, although, in this case, the swimming and climbing time were not assessed independently [60]. The other literature data regarding the behavior of *Tph2*^−/−^ mice in the FST are heterogeneous: either a reduced latency to immobility or a mild antidepressant phenotype were reported [57,58,59]. As previously discussed [60], the expression of depression-like behavior in *Tph2*^−/−^ mice in the FST may depend on specific protocols used to evaluate depression-like behavior (e.g., FST session day), age of the animals, or genetic background on which the knockouts were created. In addition, the reduction in fat content or compensatory mechanisms resulting from a life-long depletion of 5-HT signaling in the brain in *Tph2*^−/−^ mice may account for different outcomes in the FST (for review [63]).

Importantly, here we show for the first time that chronic ethanol consumption in 5-HT-depleted mice reduced immobility time and enhanced motor behavior, indicating that the 5-HT neurotransmission per se is not essential for the development of the antidepressant effect of ethanol. Additionally, it can be assumed that enhanced ethanol consumption in mice with low 5-HT function facilitated the antidepressant properties of ethanol and compensated for the negative state observed in drug-naive *Tph2*^−/−^ mice. Interestingly, similar to *Tph2*^−/−^ mice, in the Sardinian alcohol-preferring rat line, an animal model used to study alcohol dependence, an enhancement of ethanol drinking and reduction in the depression-like state were also accompanied by deficits in 5-HT function in the brain [26,76]. Thus, *Tph2*^−/−^ mice may serve as a model for the study of ethanol dependence in the future. Importantly, the increased ethanol consumption observed in mice depleted in brain 5-HT corresponds well to the characteristics of a subset of alcoholics with early-onset type II alcoholism (Cloninger type-2-like) that has also been associated with 5-HT hypofunction (for review [7]). Many common typologies, such as comorbid psychopathology (i.e., depression), antisocial phenotype, or no reactivity to SSRIs [7,58,62], have been found between *Tph2*^−/−^ mice and type II alcoholics, despite differences in the origin of impaired 5-HT transmission (*Tph2* deletion; present study vs. homozygosity for the long (L) allele for 5-HTT gene; [7]). Future studies on *Tph2*^−/−^ mice will be instrumental in discovering novel non-serotoninergic medications for the treatment of type II alcoholics.

Behavioral phenotype in *Tph2*^−/−^ mice, including enhanced ethanol consumption could be due to the adaptation mechanisms compensating for the continuous depletion in 5-HT, rather than the lack of 5-HT per se. Previously, we detected no marked differences in gene expression in the whole brain of *Tph2*^−/−^ mice compared to controls using Affymetrix [56]. Importantly, another research group has found an upregulation of 5-HT_1A_ and 5-HT_1B_ receptors in several brain regions, including the septum and frontal cortex of mice with constitutive inactivation of *Tph2* [77]. To confirm the existence of compensatory changes within the 5-HT system in our *Tph2*^−/−^ mice, further functional and gene expression analyses of defined brain structures need to be performed.

Importantly, aggression, sexual behavior, consumption of food, water, and addictive substances are associated with activation of the brain’s reward system. The reinforcement of these behaviors seen in *Tph2*^−/−^ mice ([60,61,62]; present study) may suggest an attenuated reward sensitivity [78] in response to the life-long 5-HT depletion. Indeed, constitutive inactivation of *Tph2* induced a reduction in the hippocampal dopamine [77], the key signaling molecule of the reward system [78]. Certainly, for an appropriate interpretation of the current results suggesting the hypodopaminergic state in 5-HT-depleted mice, the analysis of other components of the dopamine system, e.g., the expression level of the D_2_ receptor, as well as dopamine release in other regions of the reward system, including the prefrontal cortex, nucleus accumbens, or amygdala, should be carried out.

*Tph2* mRNA and protein are produced in cell bodies of 5-HT neurons of the brainstem raphe nuclei. It is generally accepted that slow axonal transport is responsible for the TPH2 enzyme delivery to the terminal field for the local 5-HT synthesis [79]. However, axonal *Tph2* mRNA transport to the projection areas, such as the cortex, hippocampus, striatum, hypothalamus, and cerebellum, cannot be excluded [80,81]. In this study, we analyzed the *Tph2* mRNA level in the brainstem raphe nuclei containing 5-HT-synthesizing neurons, and the hippocampus and prefrontal cortex—areas rich in 5-HT terminals and two ethanol-responsive parts of the mesocorticolimbic dopamine circuitry [82,83]. Very low levels of *Tph2* transcripts were detectable in the hippocampus and prefrontal cortex in comparison to the raphe nuclei, confirming previous studies in rats [84].

In the present work, we found that *Tph2*^+/+^ mice drinking ethanol exhibited no change in the *Tph2* transcript level in the raphe nuclei, hippocampus, and prefrontal cortex in comparison to control water-drinking animals. However, when the *Tph2* expression was compared between mice that consumed high and low levels of ethanol, we observed that *Tph2* expression in the raphe nuclei of “high ethanol-drinking” mice was 24% higher compared to “low ethanol-drinking” mice. Interestingly, at the same time, “high ethanol-drinking” animals displayed a reduced level of *Tph2* transcript in the prefrontal cortex compared to “low ethanol-drinking” mice. Differences in *Tph2* expression in the brainstem raphe nuclei and prefrontal cortex were observed only between mice that exhibited variability in the level of ethanol intake within the fourth week of ethanol exposure, but not those during the initial drug consumption. These findings may indicate that the differences in *Tph2* gene expression in the raphe nuclei and prefrontal cortex resulted from the chronic ethanol exposure rather than inter-individual traits, suggesting that modulation of the activity of TPH2 enzyme involved in 5-HT synthesis may represent a neuroplastic feedback mechanism during the development of ethanol-drinking behavior in mice. Since such a correlation was not observed in the hippocampus, it is unlikely that hippocampal *Tph2* transcripts contribute to voluntary ethanol consumption in mice. Moreover, considering a very low *Tph2* transcript level in the prefrontal cortex, it seems doubtful whether the observed decreases in *Tph2* expression in this brain region in “high ethanol-drinking” mice have any relevance to the development of ethanol-drinking phenotype.

The effects of chronic voluntary ethanol consumption on neuroadaptations within the 5-HT neurotransmission, particularly in the raphe nuclei, have been studied to a limited extent. Consistent with our findings, a recent study has demonstrated no change in *Tph2* mRNA level in the raphe nuclei of mice after chronic (for 6 weeks) 10% ethanol consumption [85]. Additionally, Popova et al. [85] have shown increases in the activity of the TPH2 enzyme and 5-HT turnover, and the transcript level (but not protein) of the 5-HT_7_ receptor in the midbrain raphe nuclei of animals after chronic ethanol intake. Importantly, no change in 5-HT content and 5-HT_1A_ receptor gene expression has been reported in the midbrain, frontal cortex, hippocampus, hypothalamus, and amygdala of mice after chronic ethanol [85]. In contrast, another group that used a progressive ethanol exposure protocol (3–10%, for 3 weeks), similar to the one we used in the current study, has found that chronic ethanol intake evoked an increase in 5-HT_1A_ autoreceptor sensitivity in the dorsal raphe nucleus of mice, suggesting a reduction in central 5-HT tone after chronic ethanol [67]. These discrepancies between studies could result from the fact that both analyses included mice, which, according to the observations in the present study, may have displayed inter-individual variations in the 5-HT response to chronic ethanol.

Previously, we have reported that a 50% reduction in *Tph2* mRNA in the whole brain resulting from the absence of one copy of the *Tph2* gene in heterozygous *Tph2*^+/−^ mice has led to about a 13% decrease in 5-HT level in the whole brain [68]. Another study on heterozygous *Tph2* mutant mice revealed about a 22% reduction in 5-HT in the rostral raphe nuclei, but not in the hippocampus, frontal cortex, or thalamus [77]. Importantly, such a decrease in the central 5-HT content did not affect the depressive, anxiety, and aggressive behavior [60,68], and ethanol-drinking behavior has not been evaluated in these mice. Other studies have shown that two mouse strains exhibiting low preference for ethanol, BALB/cJ and DBA/2A [53,54], harbored the C1473G (P447R) single-nucleotide polymorphism within the *Tph2* gene [52]. Besides this mutation, BALB/cJ mice displayed a decrease in the *Tph2* mRNA level (by ca. 20%) and the number of TPH2-immunoreactive neurons (by 28%) in the dorsal raphe nucleus, and, consequently, a reduction (by 15%) in 5-HT content in the midbrain and cortex compared to high-ethanol-preferring C57B/6J mice [53,55] that were homozygous for the high-activity 1473C allele for *Tph2* [52]. Based on these findings, lower *Tph2* transcript levels in the raphe nuclei observed in the present study in “low ethanol-drinking” mice may potentially coincide with decreased central 5-HT levels and represent an ethanol-resilient phenotype. Further studies are necessary to verify whether reduction in *Tph2* gene expression in mice consuming low levels of ethanol has any impact on TPH2 activity and/or 5-HT level.

Rats consuming high levels of ethanol exhibited blunted hypothalamic–pituitary–adrenocortical axis responses compared to low-ethanol-consuming animals [86]. On the other hand, another investigation has shown that the reduction in the glucocorticoid level induced by adrenalectomy enhanced *Tph2* gene expression in the mouse raphe nuclei [87], indicating that *Tph2* gene expression is sensitive to glucocorticoid signaling. Whether the high and low *Tph2* gene expression observed in mice in the present study is related to the fluctuation in the glucocorticoid level in the brain and plasma needs to be further assessed.

In summary, we propose that the life-long 5-HT depletion in the brain predisposes to increased ethanol consumption. However, it is not clear whether the effect is due to the lack of 5-HT per se or, rather, a compensatory response to the long-term deficiency in 5-HT synthesis. Moreover, we conclude that the absence of central 5-HT does not interfere with the antidepressant properties of ethanol. Our results indicate that regulation of *Tph2* gene expression in the raphe nuclei, and possibly prefrontal cortex, may be specifically involved in the development of ethanol-drinking behavior in mice.

Limitations of the study include the selection criteria of mice with high and low ethanol intake levels, which were due to the small group size (*n* = 17). Nevertheless, such conditions for selecting the “ethanol drinking phenotype” were sufficient to detect differences in the *Tph2* transcript levels, suggesting specificity of the observed changes. Another limitation is that we did not measure the 5-HT level within these animals.

Further more detailed molecular analysis of a larger group size and rodent lines selectively bred for high and low ethanol consumption is urgently needed to confirm the present findings regarding the importance of *Tph2* expression in development of ethanol drinking behavior. Modulation of TPH2 expression and/or activity by appropriate tools in the future will verify whether such a strategy will induce resilience to ethanol.

## Data Availability

All data supporting the conclusions of this manuscript are provided in the text and figures.
